# “Total nausea” as a manifestation of “total pain” and an expression of suffering: a case report

**DOI:** 10.1186/s13256-026-05933-z

**Published:** 2026-02-28

**Authors:** Christian Volberg, Robert Janke, Moritz Erk, Jorge Riera-Knorrenschild, Katharina Toussaint

**Affiliations:** 1 Department of Anaesthesiology and Intensive Care Medicine, Philipps University of Marburg, Baldingerstraße, 35043 Marburg, Germany; 2 Palliative Care Unit, Philipps University of Marburg, Baldingerstraße, 35043 Marburg, Germany; 3 Department of Heamatology, Oncology and Immunology, Philipps University of Marburg, Marburg, Germany

**Keywords:** Nausea, Vomiting, Palliative sedation, End-of-life care, Case report

## Abstract

**Background:**

Nausea and vomiting are common symptoms of advanced tumor diseases, which can usually be effectively managed with medication. However, in rare cases, refractory courses occur for which no structural cause can be identified, leading to severe multidimensional suffering.

**Case presentation:**

We present two cases involving white male patients, aged 64 and 61 years, with incurable metastatic gastrointestinal tumors who developed severe, therapy-resistant nausea accompanied by continuous regurgitation of saliva. In both cases, gastrointestinal transit was intact. The symptoms caused significant physical, psychological, and existential distress. An attempt was made to treat the persistent nausea and vomiting with medication. Despite using potent drugs, there was no improvement. Psycho-oncological therapy also failed to produce satisfactory results. Symptom control was achieved under sedation, and both patients died peacefully a few days later.

**Conclusion:**

These cases illustrate the concept of “total nausea,” which is analogous to the well-known model of “total pain.” Raising awareness of “total nausea” could help make future guidelines and decision-making processes for refractory symptoms at the end of life more nuanced.

## Background

Nausea affects 36% of palliative care patients at their first contact with specialized services, rising to 62% in the final 1–2 months of life and up to 71% in the final week [1]. Most affected individuals respond well to guideline-based antiemetic strategies [2]. However, in rare cases, symptoms persist despite maximum pharmacological intervention, an intact gastrointestinal passage, and the exclusion of metabolic causes. Such refractory cases in the terminal phase often result in considerable suffering, feelings of therapeutic helplessness, and ethical dilemmas [3, 4].

Cicely Saunders’ concept of “total pain” has become a guiding principle for palliative care as it provides a systematic description of the multidimensional nature of suffering, including physical, psychological, social, and spiritual aspects [5]. However, the application of this conceptual framework to non-pain-related refractory symptoms remains underdeveloped. In this context, we emphasize the importance of expanding the concept to include “total nausea”: the experience of therapy-resistant vomiting or retching that cannot be controlled pharmacologically and is significantly influenced by psychological, social, and existential factors.

On the basis of two clinical case reports of patients with metastatic gastrointestinal tumors and persistent vomiting despite adequate diagnosis and treatment, we aim to demonstrate the clinical relevance of “total nausea” and its implications for symptom control, communication processes, and palliative sedation.

## Case presentation

### Case 1: Refractory vomiting in a patient with pleural carcinomatosis following esophageal resection

A 64-year-old white male patient with a metastatic adenocarcinoma of the esophagogastric junction (AEG) tumor (ECOG 0) underwent a robot-assisted thoracoabdominal esophageal resection with a gastric pull-up following neoadjuvant chemotherapy. Although the R0 resection was successful, there was lymph node infiltration. Shortly after discharge, the patient developed a chylothorax, necessitating thoracoscopy with pleurodesis and insertion of a drainage tube. At the same time, the patient experienced persistent vomiting. Pyloric spasm was diagnosed.

Despite repeated endoscopic interventions (balloon dilation, botulinum toxin injection, and gastric peroral endoscopic myotomy [G-POEM]) over a period of 6 weeks and confirmation of an anatomically intact gastric passage, vomiting persisted. Parenteral nutrition was established as it was not possible for him to take in food orally owing to reflexive vomiting. Five-step antiemetic therapy (including metoclopramide, dexamethasone, ondansetron, fosaprepitant, and haloperidol; dimenhydrinate was not administered owing to subjective intolerance) had no discernible effect. The patient described the symptoms as a sudden reflex rather than classic nausea. As he regurgitated swallowed saliva after a short time, it was considered that conditioning as a result of weeks of vomiting was the cause. Consultations were held with colleagues from psychosomatics and psycho-oncology. However, they could not confirm a psychosomatic cause.

With psycho-oncological support and dronabinol medication to stimulate appetite, it was temporarily possible to enable oral food intake, resulting in a significant improvement in quality of life. However, pleural carcinomatosis and cytologically confirmed peritoneal signet ring cells were subsequently diagnosed, indicating recurrence of the disease. Consequently, the patient’s general condition continued to deteriorate (ECOG 4, anasarca, and hypoalbuminemia). The patient had high hopes for the palliative chemo-immunotherapy (modified fluorouracil leucovorin oxaliplatin [mFOLFOX6] + zolbetuximab) that was started on the palliative care ward 12 weeks after esophageal resection. However, owing to his poor general condition, the therapy had to be stopped after only one cycle. Second-line chemotherapy was also no longer medically justifiable.

The interdisciplinary tumor board recommended changing the treatment to best supportive care. After discussing this with the patient, vomiting resumed that same evening, even of saliva. The patient expressed a persistent desire to die over several days owing to the severe distress this caused. After receiving detailed information and being given time to consider it, he opted for continuous palliative sedation (target Richmond Agitation-Sedation Scale [RASS] −4). The vomiting ceased under propofol sedation. He died peacefully 2 days later, in the presence of his relatives.

### Case 2: Severe nausea and gagging without vomiting in a patient with osseous progression of esophageal cancer

A 61-year-old white male patient with a history of robot-assisted subtotal esophagectomy (R0 resection, N1 M0, without neoadjuvant or adjuvant therapy at the patient’s explicit request) presented with osseous metastasis of his esophageal carcinoma 2 years postoperatively. Owing to multiple pathological fractures and fracture-prone osteolysis (in the pelvis and femur), the patient was admitted to the palliative care unit for pain management. The patient strictly refused chemotherapy but agreed to analgesic radiation therapy. He lived alone, was cachectic (ECOG 3), and wanted to return to work but consistently refused nursing support.

Analgesia was first administered intravenously with morphine and subsequently via transdermal fentanyl. Oral medication was rejected owing to nausea induced by tablets. Despite receiving adequate pain therapy, mobilization was difficult owing to the risk of femur fracture, and surgical treatment was not an option given the patient’s poor general condition. Returning to work was therefore not an option. Realizing this, the patient developed severe nausea with a constant gag reflex but no actual vomiting. Comprehensive antiemetic escalation involving transdermal ondansetron, as well as intravenous haloperidol, dexamethasone, metoclopramide, dimenhydrinate, and fosaprepitant remained ineffective. After a few days, the patient refused any form of intravenous therapy and expressed an increasing desire to die. He also refused psycho-oncological support.

After receiving the news of the deaths of two close colleagues from his professional environment, his symptoms worsened noticeably, with him constantly gagging and retching up saliva. Given his now extremely high level of multidimensional suffering, the patient explicitly requested palliative sedation. After being fully informed and providing his consent, sedation was initiated (target RASS −4). With continuous propofol sedation, his gag reflex ceased completely. The patient died peacefully 3 days later on the palliative care unit.

## Discussion and conclusion

The two cases described above clearly demonstrate the challenges that palliative care providers face in managing refractory gastrointestinal symptoms that cannot be controlled either causally or symptomatically. Despite adequate diagnostics, escalation of antiemetic medication, and interdisciplinary support, adequate symptom control remained unattainable in both cases.

Most studies on emesis in advanced tumor diseases focus on pharmacological strategies [2, 3], while the question of whether end-of-life vomiting and retching reflects multidimensional suffering akin to Saunders’ “total pain” model is rarely addressed [5].

The term “total nausea” precisely describes this form of suffering: a multidimensional condition involving nausea, retching, and vomiting, where the symptoms take on a life of their own and become an expression of existential overload. Conventional approaches are no longer effective in treating this condition. To date, the term has hardly been conceptualized in international literature [6], only case reports and individual narrative contributions point in this direction [7]. Similar to “total pain,” “total nausea” encompasses physical, psychological, social, and spiritual components. These aspects can also be observed in the two cases described:

Physical: Cancer of the upper gastrointestinal tract was diagnosed in both patients.

Psychological: A psychosomatic cause, such as anticipatory nausea, could be ruled out. Patient 1 was open to psychological interventions, whereas patient 2 refused counseling.

Social: The patient in the first case report left his wife and children behind, while the patient in the second report suffered a significant worsening of symptoms when he learned that two work colleagues who meant a lot to him had died.

Spiritual: Both patients had high hopes of continuing to live and had not come to terms with the end of their lives.

This concept is supported by studies on anticipatory and conditioned nausea. Roscoe et al. demonstrated that nausea can become reflexive, a phenomenon that appears to be applicable to terminal illnesses [8]. However, anticipatory nausea is a learned response to an expected stimulus, which can be unlearned and responds better to therapy than “total nausea.” To investigate this possible cause and determine whether treatment was necessary, colleagues from the departments of psychosomatics and psycho-oncology were consulted. However, anticipatory nausea was ruled out, meaning that no psychological interventions could be carried out in this regard. Henson et al. also highlight the interaction between nausea, loss of autonomy, and psychosocial stress [9]. Qualitative studies also describe “inner suffocation” as an expression of terminal overload due to uncontrollable vomiting. Thus, “total nausea” could be considered the experienced chaos of the body, mind, and surroundings [10]. In both cases described above, attempts at psycho-oncological cognitive restructuring, brief interventions, and discussions about coping with and accepting the disease were unsuccessful, as the patients continued to experience nausea and vomiting.

In both cases, the decision to administer palliative sedation was made in accordance with the European Association of Palliative Care (EAPC’s) ethical guidelines and in agreement with the patients and their families. The therapeutic focus shifted from control to relief, as a last resort to alleviate suffering [11]. In retrospect, both cases appear to exemplify symptoms that are difficult to grasp using conventional terms and guidelines.

Comparing the German S3 guideline, Palliative Medicine for Patients with Incurable Cancer, and the US National Comprehensive Cancer Network (NCCN) guideline, Palliative Care, shows that both documents provide differentiated recommendations for the pharmacological treatment of nausea. However, neither offers a structured approach to capturing the complex, multidimensional nature of nausea as documented in the present case reports. Both guidelines acknowledge that psychosocial factors, such as anxiety, expectations, and existential stress, can influence nausea. The German guideline emphasizes that therapy-resistant nausea should be assessed on an interdisciplinary basis, including a psychosomatic evaluation. The NCCN guidelines clearly distinguish between anticipatory, chemically induced, and refractory nausea, and they incorporate nonpharmacological approaches such as behavioral therapy and aromatherapy [12, 13].

However, both guidelines lack an explicit conceptual framework, such as the term “total nausea” formulated by Darrah and Goldberg, which provides an integrative view of the physical, psychological, social, and spiritual factors that influence the symptom as an overall phenomenon [7]. This gap is particularly evident when considering the clinical courses of the case reports presented, in which classic antiemetic strategies failed despite maximum drug therapy, and the experience of nausea transcended purely physical explanatory models. The guidelines could address this issue in more detail in the future, but further research on the topic is needed to enable sustainable recommendations to be made.

In both patients, continuous propofol sedation resulted in immediate and complete cessation of nausea, gagging, and regurgitation. Symptom control was maintained until death, which occurred peacefully after 2 and 3 days, respectively, in the presence of relatives. No additional complications were observed.

These cases highlight the need to establish “total nausea” as a distinct clinical concept and to integrate it into clinical guidelines and decision-making processes. To achieve this, prospective registries and qualitative studies are required to capture its multidimensional nature and provide a systematic framework for assessment and treatment. Such research could also reduce diagnostic uncertainty, facilitate earlier identification of affected patients through assessment tools, and improve interdisciplinary communication. Furthermore, future work should clarify whether existing palliative sedation guidelines sufficiently address refractory gastrointestinal symptoms and how they may be expanded to meaningfully include this complex phenomenon.

## Key statements

- Refractory nausea and vomiting without a structural cause are independent, multidimensional symptoms at the end of life that sometimes cannot be controlled by medication and cause severe distress.
- The concept of “total nausea” complements existing models, such as “total pain,” by integrating physical, psychodynamic, social, and existential factors into the perception of nausea and vomiting.
- Within the framework of “total nausea,” continuous palliative sedation can be an ethically and medically justifiable option for treating refractory gastrointestinal symptoms when all other curative and symptomatic options have been exhausted.

## Data Availability

Data sharing is not applicable to this article as no datasets were generated or analyzed during the current study.

## Abbreviations

AEG tumor: Adenocarcinoma of the esophagogastric junction
ECOG: Eastern Cooperative Oncology Group
G-POEM: Gastric peroral endoscopic myotomy
mFOLFOX6: Modified fluorouracil leucovorin oxaliplatin
RASS: Richmond Agitation-Sedation Scale
EAPC: European Association of Palliative Care
NCCN: National Comprehensive Cancer Network
